# Quality of the Exotic Parasitoid *Cotesia flavipes* (Hymenoptera: Braconidae) Does Not Show Deleterious Effects after Inbreeding for 10 Generations

**DOI:** 10.1371/journal.pone.0160898

**Published:** 2016-08-10

**Authors:** Maíra Trevisan, Sergio A. De Bortoli, Alessandra M. Vacari, Valéria L. Laurentis, Dagmara G. Ramalho

**Affiliations:** 1 Laboratory of Biology and Insect Rearing (LBIR), Department of Plant Protection, São Paulo State University—FCAV/UNESP, Jaboticabal, São Paulo, Brazil; 2 Department of Biology, São Paulo University–USP, Ribeirão Preto, São Paulo, Brazil; CNRS, FRANCE

## Abstract

Although the parasitoid *Cotesia flavipes* (Cameron) has proven effective in controlling sugarcane borer *Diatraea saccharalis* (Fabricius) for many years, concern has arisen over the quality of individuals produced at large scales. The parasitoid has been reared in laboratories in Brazil for more than 40 years, with no new introductions of new populations during that period. Since the quality of the parasitoids was not verified at the time of the species' introduction in Brazil, we do not know if there has been any reduction in quality so far. However, it is possible to determine whether the parasitoid could reduce in quality in future generations. Thus, the objective of this research was to assess the quality of these insects over 10 generations and look for evidence of any loss in quality. We used two populations: one from a biofactory that has been maintained in the laboratory for over 40 years, and an inbred laboratory population. Both were bred, and compared for 10 generations. We wanted to determine what happened to the quality of the parasitoid after 10 generations in an extreme inbreeding situation. To assure inbreeding, newly emerged females were forced to mate with a sibling. Individual females were then allowed to parasitize larvae of *D*. *saccharalis*. We performed evaluations for each generation until the tenth generation, and recorded the sex ratio, percentage emergence, number of offspring/females, and longevity of both males and females. Results of the measurements of biological characteristics demonstrated random significant differences between populations; best results were obtained intermittently for both the biofactory population and the inbred population. No significant differences across generations for the same population were observed. Thus, rearing of a *C*. *flavipes* population subjected to inbreeding for 10 generations was not sufficient to reveal any deleterious effects of inbreeding.

## Introduction

Brazil is the world leader in sugarcane production. Responsible for over half of the sugar traded in the world, the country has approximately 8.8 million hectares planted with an production about of 672 million tons of sugarcane in 2014/2015 [1]. This amount of sugarcane can produce 32.5 million tons of sugar and 25.87 billion liters of ethanol [2].

As with any crop, sugarcane has a variety of insects that are associated with its production. Of several insect species that can cause damage, the sugarcane borer *Diatraea saccharalis* (Fabricius, 1794) (Lepidoptera: Crambidae) is notable; it is a lepidopteran whose larval form opens galleries inside the sugarcane stalk [3]. Its ability for damage is compounded by the frequency with which it occurs in the cane fields, its high biotic potential, and the favorable climate in Brazil [4]. The direct damage caused by the larvae of *D*. *saccharalis* is related to the attack on sugarcane plants caused by its feeding. Galleries formed within the stem weaken the plant, making it easier to fall, and eventually can lead to death or breakage, a symptom known as "dead heart" [5].

Indirect losses occur through the colonization of fungi, which cause red stem rot diseases. The holes left by the opening of the galleries facilitate the entry of microorganisms such as *Fusarium moniliforme* and *Colletotrichum falcatum*. These organisms cause the inversion of sucrose and decrease the purity of the broth, which damages both the agricultural and industrial crop yield [3].

Because of the biology of the pest and the extensive continuous areas cultivated with cane sugar, chemical control of the sugarcane borer is inefficient. It spends most of its larval stage inaccessible to contact with insecticides, and chemical control would also be costly, depending on the size of the crop, and potentially harmful to the environment [6].

The parasitoid *Cotesia flavipes* (Cameron, 1891) (Hymenoptera: Braconidae) was successfully introduced in Brazil beginning in 1974 [7, 8] and from 1980 to 2002 the intensity of infestation of this pest decreased from 11% to 2.8% [9]. Although the parasitoid has proven effective in controlling the borer, currently there is concern regarding the quality of mass-produced parasitoids [10, 11].

Confidence in the effectiveness of a biological control agent is vital for a biological control program to become established and be successful. Unsatisfactory results caused by poor quality of control agents may result in negative publicity for the method and jeopardize a program in which many years of research were invested [12]. The success of entomophagous control agents, which may keep the populations of pests in check, largely depends on efficient methods of mass production, field release, and the potential of the species to reduce the pest population [13].

One of the biggest obstacles to quality control is the detection of loss of genetic variability in insects reared in large quantities. Considering all the mechanisms that can cause a loss in variability, inbreeding appears to be the most relevant to established colonies in laboratories [14, 15]. Inbreeding is defined as matings between related individuals, including crosses between siblings, parents and offspring, and between cousins [16]. The resulting offspring may display in each generation a higher frequency of homozygous recessive alleles, which may carry deleterious or otherwise undesirable characteristics [17].

The consequences of inbreeding are reflected in the loss of genetic variability, and they can influence the size of the insects, the viability and fertility of the offspring, juvenile and adult mortality, as well as the morphology of individuals [18]. Alteration of these factors may compromise the effectiveness of the biological control agent in the field [19].

In the current Brazilian situation, populations were founded by a small group of individuals with no further reports of wild strains being introduced. Although mating is random within the populations grown in biofactories, it does not result in increased genetic diversity because all individuals are related, having some common ancestors [20]. Thus, one can consider the Brazilian population as a single population of *C*. *flavipes*, even if divided into sub-populations in different biofactories.

Since its introduction in Brazil, the quality of parasitoids has not been monitored; therefore, we do not know if that species lost quality over time, but we could know whether during the next generations the parasitoid will lose quality or not. Thus, the objective was to determine the quality of these insects over the next 10 generations and if there is evidence of quality loss. Therefore, we forced matings between *C*. *flavipes* siblings to determine if there is deterioration or lose of aggressiveness of parasitoids over the next 10 generations. Our research will help to clarify what has been happening to Brazilian populations of *C*. *flavipes* being reared in laboratories for >40 years without introduction of new parasitoids.

## Materials and Methods

Our research was performed at the Laboratory of Biology and Insect Rearing (LBIR) of the Plant Protection Department of the Universidade Estadual Paulista "Julio de Mesquita Filho"—Campus Jaboticabal, São Paulo, Brazil. The insects were kept in temperature-controlled room at 26 ± 1°C, 70 ± 10% relative humidity, and a 12L:12D photoperiod.

### Obtaining Specimens

The required biological material for the experiments was obtained from the Laboratory of Entomology of Usina São Martinho, located in Pradópolis, São Paulo, Brazil, which provided *D*. *saccharalis* larvae in the third instar, artificial diet for caterpillars, and cocoon masses of *C*. *flavipes*.

### Rearing and Inbreeding the Population

Rearing of inbred individuals began with the specimens obtained from the Laboratory of Entomology of Usina São Martinho. Masses of cocoons were taken to LBIR, and as soon as individuals emerged, the adults from the same cocoon mass were separated into couples. The immediate separation of couples ensured that each unmated female was isolated with a single brother, thereby ensuring that the offspring presented the genetic characteristics of that couple. Next, the mated females were allowed to parasitize larvae of *D*. *saccharalis*. Likewise, the descendants of these couples were also forced to mate only with a sibling and the female subsequently parasitized other caterpillars. This was repeated generation after generation, thereby evaluating the effects of inbreeding on the efficacy of parasitism by *C*. *flavipes*.

The experiments were performed for 10 generations of the parasitoid *C*. *flavipes*. Individuals from the mass production of Usina São Martinho (control) and individuals obtained from crosses between siblings were compared. Each new generation was evaluated, as well as a new control group.

### Biological Characteristics

After adult emergence, 50 pairs of the inbred population were removed. Each pair was placed in a Petri dish (6 cm diameter × 2.5 cm) without food. After 24 h, a *D*. *saccharalis* caterpillar in the third instar was offered to each female, and the parasitized caterpillar was stored in Petri dishes with artificial diet; the dish was marked to identify the female that parasitized it.

Each parasitized caterpillar was considered a replicate. Caterpillars were kept under controlled conditions for approximately 5 days until the formation of the pupae of *C*. *flavipes* on the host body. *C*. *flavipes* cocoons were in kept Petri dishes, identifying their origin, until the emergence of adults. Fifty samples of cocoon masses from the biofactory population of parasitoid cocoons were reserved from the same generation as that of the inbred population to serve as the control. They were maintained under the same conditions of temperature, photoperiod, and relative humidity as the test subjects.

The adult offspring obtained from each replicate of the treatment and control populations were counted, along with the viable pupae, to determine the percentage of adult emergence and sex ratio. This procedure was conducted for each generation until the tenth generation.

### Adult Survival

To evaluate adult survival rate, 20 newly emerged couples from both populations were removed from each generation. Individuals were kept in flat-bottomed test tubes (2 cm diameter × 8 cm height) that were sealed with plastic wrap. The wasps were kept without food, and were observed every eight hours from emergence until death (cessation of movement). The survival assessments for males and females were performed at each generation, up to the tenth, for the two populations of the parasitoid.

### Data Analysis

The effects of the different parasitoid populations were analyzed using the repeated measures procedure for an analysis of variance (ANOVA; Proc Mixed). Data from biological characteristics (pupal period, percentage of emerged adults/mass, total of emerged adults/mass, and sex ratio) were submitted to this analysis. Each biological characteristic was analyzed separately (independent fixed variables, treatment and time; random variable, and replicates within treatment), and an appropriate covariance structure for each characteristic was used [21]. As there was a significant interaction between the main effects (populations and generations), an additional analysis of variance was performed for each treatment. Assumptions of normality and homogeneity of variance were checked using the Cramer-von Mises criterion and Bartlett’s test. If significant differences were found between the treatments, means were compared using Tukey’s test. Moreover, curves were drawn using data for the age-specific survival and were compared according to Kaplan and Meyer [22].

## Results

### Biological Characteristics

The pupal period of *C*. *flavipes* was significantly different between generations (*F*_10, 1011_ = 212.63, *P* < 0.0001) and populations (*F*_1, 1011_ = 699.31, *P* < 0.0001), in which individuals of the inbred population had a longer pupal period (6.2 days). Moreover, a difference occurred in the populations x generations interaction (*F*_9, 1011_ = 115.59, *P* < 0.0001). The significant interaction indicated that the pupal period of *C*. *flavipes* populations studied varied with the generation of the insect.

The F1 generation of the inbred population had a pupal period of 3.9 days, in contrast with the F4 generation, which had a longer duration of 7.8 days (*F*_10, 539_ = 168.07, *P* < 0.0001). For the cocoons derived from the biofactory, pupal period was also different and ranged from 3.8 days in the F3 generation to 7.0 days in F8 generation (*F*_9, 490_ = 156.90, *P* <0.0001) (Table 1).

**Table 1:** Pupal period of individuals from two populations of *Cotesia flavipes*.

	Population	
	Inbred	Biofactory
P	6.0 ± 0.00 f^1^	-
F1	3.9 ± 0.03 h	5.0 ± 0.00 c*
F2	5.4 ± 0.15 g	6.0 ± 0.00 b*
F3	6.0 ± 0.00 f*	3.8 ± 0.05 f
F4	7.8 ± 0.09 a*	5.9 ± 0.13 b
F5	6.4 ± 0.08 cd*	4.6 ± 0.07 d
F6	6.1 ± 0.06 ef*	5.1 ± 0.10 c
F7	6.1 ± 0.07 def	5.9 ± 0.08 b
F8	7.4 ± 0.09 b*	7.0 ± 0.00 a
F9	6.6 ± 0.07 c*	5.1 ± 0.11 c
F10	6.4 ± 0.08 cde*	4.2 ± 0.11 e

^1^Means ± standard error

*denotes difference in the rows, and lowercase letters indicate differences in the column (P < 0.05).

The percentage of emerged adults of *C*. *flavipes* was different between generations (*F*_10, 1011_ = 19.41, *P* < 0.0001), but not between populations (*F*_1, 1011_ = 1.24, *P* = 0.2650). The highest rate of emergence was observed in the F4 generation (42.1%). Moreover, there was a difference in the populations x generations interaction (*F*_9, 1011_ = 3.64, *P* = 0.0002).

The percentage of adults emerged from pupae in the mass of the population biofactory showed values ranging from 59.5% in the F1 generation (32.3 insects/mass) to 80.8% in the F9 generation (54.9 insects/mass) (*F*_9, 470_ = 13.82, *P* <0.0001). For the inbred population, the percentage of emerged adults ranged from 57.6% in F8 generation (37.1 insects/mass) to 81.9 in the F2 generation (57.9 adults/mass) (*F*_10, 539_ = 15, 59, *P* < 0.0001) (Tables 2 and 3).

**Table 2:** Percentage of adults emerged per mass from two populations of *Cotesia flavipes*.

	Population	
	Inbred	Biofactory
P	70.9 ± 0.00 abc^1^	-
F1	71.6 ± 2.89 ab*	59.5 ± 2.96 c
F2	81.9 ± 1.99 a*	72.6 ± 2.65 ab
F3	74.8 ± 2.74 ab	73.8 ± 2.58 ab
F4	38.1 ± 3.69 d	66.9 ± 4.55 abc*
F5	69.6 ± 3.07 abc	71.7 ± 3.00 abc
F6	62.9 ± 3.18 bc	66.3 ± 2.99 bc
F7	67.2 ± 2.83 bc	72.2 ± 3.02 abc
F8	57.6 ± 3.32 c	75.5 ± 1.80 ab*
F9	75.9 ± 2.71 ab	80.8 ± 2.02 a
F10	62.9 ± 2.89 bc*	43.9 ± 3.22 d

^1^Means ± standard error

*denotes difference in the rows and lowercase letters indicate differences in the column (P < 0.05).

**Table 3:** Total of adults emerged per mass from two populations of *Cotesia flavipes*.

	Population	
	Inbred	Biofactory
P	57.0 ± 3.35 a^1^	-
F1	50.5 ± 3.19 ab*	32.3 ± 2.31 b
F2	57.9 ± 3.24 a	56.8 ± 3.97 a
F3	55.5 ± 3.53 a	49.9 ± 3.73 a
F4	24.4 ± 2.73 c	30.2 ± 4.16 b
F5	55.5 ± 3.86 a	57.2 ± 3.76 a
F6	43.9 ± 3.85 ab	42.5 ± 3.87 ab
F7	55.9 ± 3.64 a	54.6 ± 4.25 a
F8	37.1 ± 2.65 bc	58.3 ± 3.17 a*
F9	44.7 ± 3.12 ab	54.9 ± 2.32 a*
F10	42.7 ± 3.42 ab*	31.5 ± 2.97 b

^1^Means ± standard error

*denotes difference in the rows and lowercase letters indicate differences in the column (P < 0.05).

The emerged adults were separated by sex and it was observed there were more females in all generations reared in the inbreeding group (*F*_10, 539_ = 5.77, *P* < 0.0001). In F1, F5, and F7 the number of females was significantly higher in the population reared in inbreeding when compared to the values of the respective generations of the population reared in biofactory (Table 4). However, the overall average sex ratio was similar for the two populations, being 0.64 in the inbred populations (*F*_10, 539_ = 5.77, *P* < 0.0001) and 0.61 in the biofactory population (*F*_9, 471_ = 6.18, *P* < 0.0001).

**Table 4:** Sex ratio of individuals from two populations of *Cotesia flavipes*.

	Population	
	Inbred	Biofactory
P	0.8 ± 0.00 a^1^	-
F1	0.7 ± 0.02 abcd*	0.5 ± 0.05 cd
F2	0.8 ± 0.01ab	0.7 ± 0.03 ab
F3	0.7 ± 0.02 abcd	0.7 ± 0.04 abc
F4	0.7 ± 0.03 bcd	0.8 ± 0.05 a
F5	0.7 ± 0.03 bcd*	0.6 ± 0.04 bcd
F6	0.6 ± 0.04 d	0.5 ± 0.04 d
F7	0.7 ± 0.02 bcd*	0.5 ± 0.05 d
F8	0.6 ± 0.04 cd	0.5 ± 0.03 cd
F9	0.7 ± 0.03 bcd	0.6 ± 0.03 abcd
F10	0.7 ± 0.02 abc	0.7 ± 0.04 abc

^1^Means ± standard error

*denotes difference in the rows and lowercase letters indicate differences in the column (P < 0.05).

### Adult Survival

**Fig 1:**
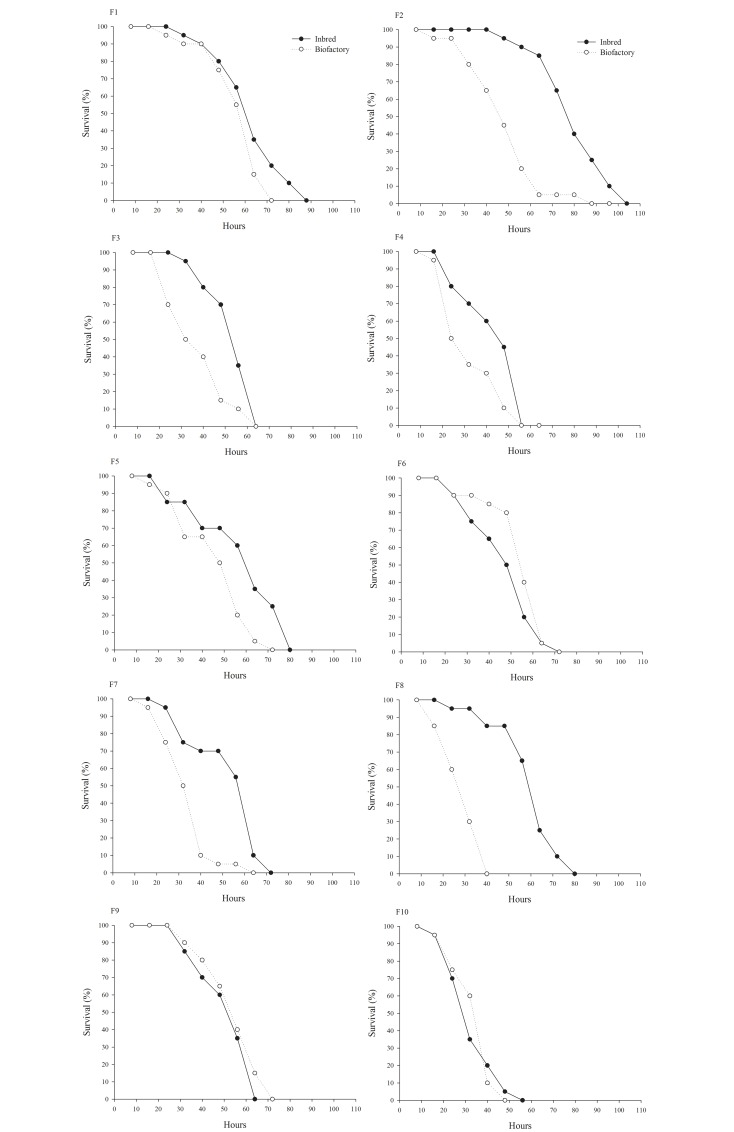
Male survival of *Cotesia flavipes* from a population subjected to inbreeding and a control population.

The males in the F2 generation of the inbred population survived longer (Fig 1). After 65 hours, only 5% of the individuals from the biofactory population were still alive, whereas in the same period the survival of the inbred population was 80% (GL = 1, χ² = 10.5503, *P* = 0.0012).

For the survival of males in the F3 generation, at approximately 50 hours, the percentage of individuals living in the biofactory population was 15%, whereas the that of the inbred population, in the same period, was about 80% of (GL = 1, χ² = 11.0585, *P* = 0.0009). However, there was a sharp drop in the percentage of live adults in the inbred population between 50th and 65th hours, and death of 100% of individuals after 65 hours occurred for both populations (Fig 1).

In the F7 generation, the survival of male subjects was also different (GL = 1, χ² = 12.5116, *P* = 0.0004), at approximately 50 hours, the percentage of survivors was 80% in the inbred population, whereas during the same period only 5% survived in the biofactory population (Fig 1).

The biggest difference in survival was observed during the F8 generation between the populations (GL = 1, χ² = 34.9683, *P* < 0.0001). After 40 hours, 100% of the male population of the biofactory population was dead, whereas 80% of the inbred individuals were still alive. For the inbred population, 100% mortality was observed after 80 hours (Fig 1). In F1, F6, F9, and F10, there was no significant difference in the survival rates for male individuals between populations (Fig 1).

**Fig 2:**
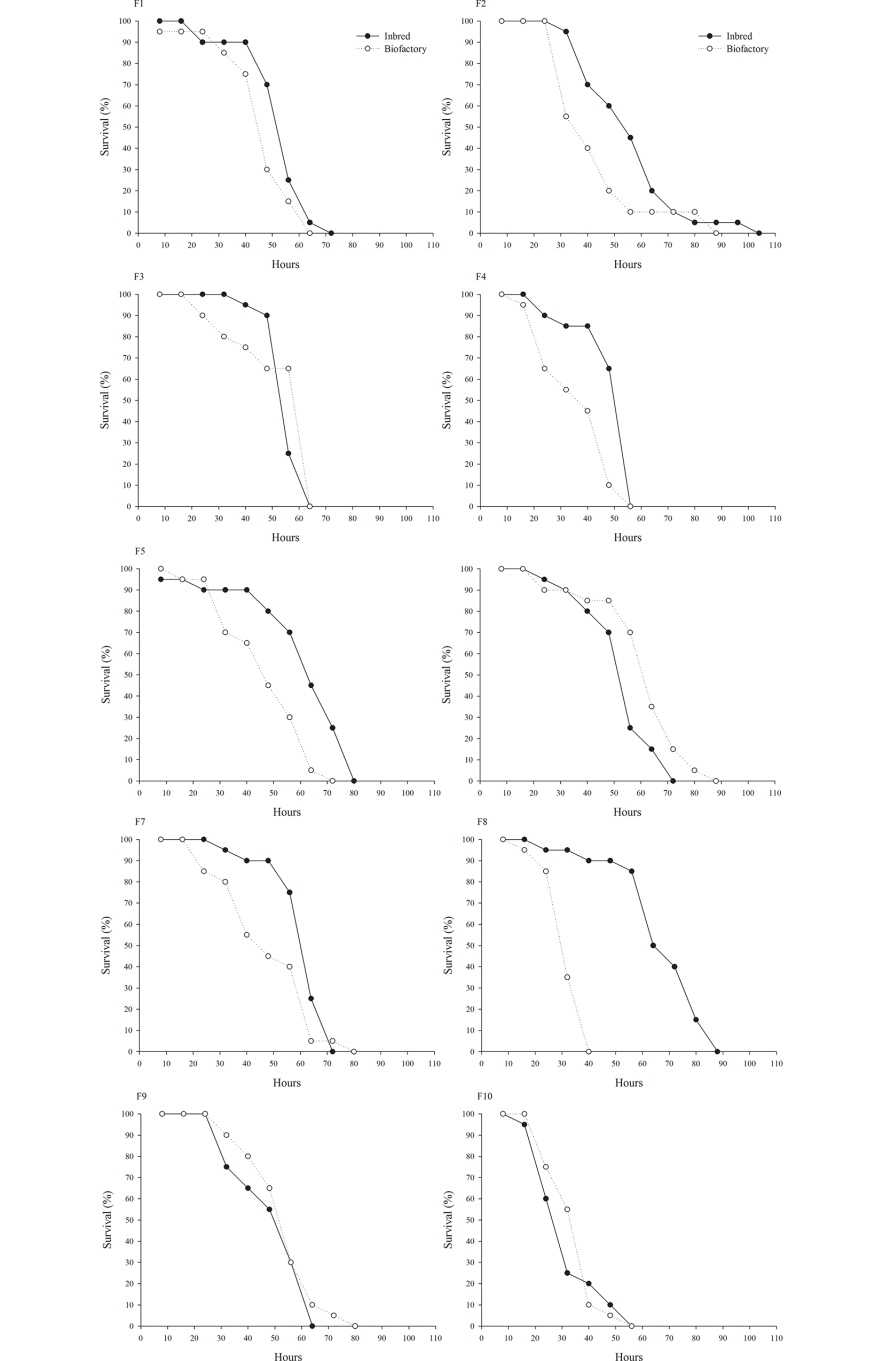
Female survival of *Cotesia flavipes* from a population subjected to inbreeding and a control population.

Regarding the survival of females, difference in the F2 generation was observed (GL = 1, χ² = 6.0395, *P* = 0.0140) (Fig 2), with a decrease in the percentage of survivors from 100% to 50% for the biofactory population between 25 and 35 hours. After 90 hours, mortality was recorded for all individuals. In the inbred population, 50% of survivors were observed at 60 hours and mortality for all individuals at 115th hour.

In the F4 generation, although the percentage of live females from the inbred population was close to 85% at 40 hours for the females, in the biofactory population it was 50% (GL = 1, χ² = 13.9707, *P* = 0, 0002). The mortality of all females was observed after 55 hours in both groups (Fig 2).

Again the F8 generation exhibited the greatest discrepancy in relation to survival of females between populations (GL = 1, χ² = 34.8670, *P* < 0.0001). Thus, while 100% of the females from the biofactory population were already dead after 40 hours, 90% of the female from the inbred population survived, and only showed 100% mortality after 90 hours.

## Discussion

The severity with which the effects of inbreeding are exhibited in a population is closely related to the reproduction type of a species [23]. In haplodiploides groups such as Hymenoptera, the effects of loss of genetic diversity through inbreeding appear to be mild because of the genetic buildup of genders [23]. As the females of this group are heterozygous for the locus responsible for sex determination and males are hemizygous for the same locus, the recessive genetic load hidden in heterozygosity of the female is completely expressed by the male. Thus, the deleterious recessive alleles expressed by males might be subject to the selection of the environment and eliminated from the population, thereby reducing the deleterious effects of inbreeding [24, 25, 26].

Henter [23] gathered 45 studies from 25 different species both diploid and haplodiploides, and by means of a meta-analysis, evaluated the differences of the effects of inbreeding in these two groups. The results showed that the effects of inbreeding depressions are more severe in survival and fecundity of adult diploid species than in haplodiploides species. Werren [25] suggested that under ecological conditions requiring the occurrence of inbreeding, such as low population density, haplodiploides species are more likely to survive the transition from outbreeding to inbreeding than diploid species.

Besides the advantage offered by haploidiploidia in eliminating deleterious alleles, the Hymenoptera employ a wide variety of mating systems, ranging from restricted crossings between siblings to random mating between individuals of the population [27]. A low-level genetic inbreeding depression is naturally expected in inbred populations because the wide expression of deleterious alleles allows for the elimination of these genes from the population [23].

The distribution of hosts and the habits of the larval parasitoid have great influence on the frequency with which inbreeding can occur naturally. According to Godfray [28] and Henter [23], it is expected that gregarious species of parasitoids present a higher frequency of inbreeding than solitary species. A good example is seen in *C*. *glomerata* (Linnaeus, 1758) (Hymenoptera: Braconidae), whose natural host (*Pieris brassicae*) exhibits an isolated distribution and the occurrence of inbreeding is not uncommon for this parasitoid [29]. The males of this species typically emerge earlier than females, and approximately 70% of all males remain near the site of emergence [30], hoping to mate with their female siblings [31]. Arakaki and Ganaha [32] observed that *C*. *flavipes* also has high rates of inbreeding after its emergence and Niyibigira et al. [33] suggested that this behavior occurs regularly in the species.

Another condition that makes outbreeding more likely is the polygamous nature of the female. At least 2/3 of the gregarious parasite species listed by Ridley [34] depict cases of the female accepting copulation with more than one male. This can easily occur on a regular basis for *C*. *flavipes* in the biofactory laboratory, where about 15 cocoons masses from different females are put together until the moment of release of the adults in the field [35]. Progenies generated with different genetic loads have better genetic variety even if siblings mate.

However, gregarious species with high frequencies of inbreeding in the field may still suffer the effects of an inbreeding depression, such as *Trichogramma pretiosum* (Riley, 1871) (Hymenoptera: Trichogrammatidae) [36, 26]. Although the deleterious recessive alleles can be removed efficiently by haploid males, alleles that carry slight deleterious traits or that present traits of partial dominance are more difficult to remove from the population and may cause the permanence of an inbreeding depression in haplodiploides insects [37].

The effects of inbreeding depressions may be reflected in the parameters used in the evaluation of quality control of the parasitoids, such as longevity [19]. However, its effects can be measured mainly by characteristics limited to females, such as fecundity, looking for hosts, and determining the sex ratio of offspring [23, 26].

Overall, the present results concerning the biological characteristics of both sexes are similar to those obtained in other studies. The pupal period of the two populations studied was similar to that observed by Hernández [38] (5.4 days) and Zhou et al. [39], who found no significant differences in development time of *C*. *glomerata* between inbred and outbred groups. In studies conducted in Texas, [40] expected to find effects of inbreeding in a colony of *C*. *flavipes* reared more than four years in the laboratory with no new introduction of wild individuals. However, when the results were compared with data from two other groups, reared a shorter period of time in the laboratory and from other regions (a group from another region of Texas and another from Thailand), the differences were minimal.

Regarding the characteristics expressed only by females, Henter [23] noted that after only five generations under inbreeding, the solitary wasp *Uscana semifumipennis* Girault, 1911 (Hymenoptera: Trichogrammatidae) showed a decrease in fecundity and sex ratio of offspring as a consequence of an inbreeding depression. Zhou et al. [39] evaluated the effect of inbreeding depression in *C*. *glomerata* and observed that in the generation F1 of the inbred group, the production of males was significantly higher (43%) than in the outbred group (28%) and at the fourth generation the percentage of male progeny already exceeded 70%. Another braconidae evaluated under inbreeding was *Asobara tabida* (Nees 1834) (Hymenoptera: Braconidae), in which the size of the offspring and the proportion of females were higher in the inbred compared with the outbred group [41].

The increase in the proportion of males leads to a decrease in the rate of population growth, with a consequent rise in the potential for extinction of the population [42]. Thus, an increase in males is considered a very serious effect of an inbreeding depression. Male production can increase significantly after successive consanguineous matings as a result of the action of inbreeding on the mechanism of sex determination of the species. There are four different models of sex determination proposed for the Hymenoptera [43]. The most common, known as complementary sex determination (CSD) [44], has two variations according to the number of loci involved. When a single locus is responsible for determining the sex, the mechanism is called a single locus-CSD (sl-CSD) [45]. When sex is determined from multiple loci, it is called multiple loci-CSD (ml-CSD) [46, 47].

The sl-CSD occurs in almost all superfamilies of Hymenoptera, with the exception of Chalcidoidea [48], leading to the belief that this type of sex determination is the ancestral mechanism within the group [49]. Under sl-CSD, the sex of an individual is determined by the allelic composition at sexual locus. Haploid individuals present only one allele in the sex locus and are always males. Diploid individuals have two different alleles and may be female, if the two copies of the alleles are different, or they may be male if the copies of the alleles are equal [44]. If the sl-CSD mechanism applies to an inbreeding species where male and female share a sex allele, the proportion of males in the progeny will be higher because 50% of the fertilized eggs will become diploid males [44]. In general, diploid males exhibit low viability [43, 45, 50], an inability to mate [51], or are sterile [52] because the sperm is unable to penetrate the egg [53]. Occasionally, some manage to carry out fertilization, but produce sterile triploid daughters [54]. Thus, the occurrence of diploid males impairs the production of females in the next generation, increasing the proportion of males in each generation and decreasing the number of descendants.

Empirical studies do not support the hypothesis that sl-CSD is the only mechanism of sex determination in the genus *Cotesia* [33, 44, 55], which includes about 100 species worldwide [56]. Until 2006, the only species of the genus *Cotesia* for which its mechanism of reproduction was determined to be sl-CSD was *C*. *glomerata* [39]. Field populations of *Cotesia sesamiae* (Cameron, 1906) and *Cotesia rubecula* (Mason, 1981) (Hymenoptera: Braconidae) showed no evidence of sl-CSD [33, 42].

Sl-CSD species often have mechanisms to prevent the occurrence of mating between relatives [48]. Behaviors in adults after emergence for this purpose were observed for *C*. *glomerata* and *Bracon hebetor* (Say, 1836) (Hymenoptera: Braconidae). Over 50% of females of *C*. *glomerata* and 30% of males were observed leaving their place of birth before mating [30]. For *B*. *hebetor*, both males and females are not receptive to mating for the first two hours after emergence [57].

However, under the ml-CSD system, in order for an individual to develop as a diploid male, he must be homozygous in a number of loci. Thus, there must be many inbreeding generations in order to have a considerable production of diploid males [33]. Crozier [58] suggested that in species exhibiting ml-CSD, the occurrence of diploid males remains rare even under inbreeding, since occasional outbred crosses would be sufficient to restore heterozygosity at some of the sex loci.

The genus *Cotesia* apparently presents a variety of solutions to the problem that arises with the production of diploid males: (1) having reproductively functional diploid males as *C*. *glomerata* [59]; (2) presenting the ml-CSD mechanism and reducing the frequency of diploid males, as in *C*. *vestalis* (Haliday, 1834) (Hymenoptera: Braconidae) [60] and *C*. *rubecula* [61]; and (3) do not exhibiting the CSD mechanism and thus, completely preventing the production of diploid males, as *C*. *flavipes* [33].

We found no increase in the proportion of males over 10 generations of *C*. *flavipes*. Similar results were observed by Niyibigira et al. [33], who found no increase in the proportions of males or any hint of an inbreeding depression even after 25 generations of *C*. *flavipes* reared in inbreeding conditions, suggesting that this species has a different type of sex determining mechanism, with the "imprinting" being the most likely. In this type of mechanism, the sex locus (X) binds to an active product in the egg or zygote. During the process of meiosis, the female transfers her sexual locus with a brand. In unfertilized eggs, that mark does not allow the locus to link to the active product, making it inactive and this mark is erased in males during embryonic development. Without this tag, the paternal allele is able to synthesize the active product. Therefore, in fertilized eggs, the sexual locus receives a tagged allele from the mother and one unmarked allele from the father, activating the link on the locus X with the active product, so that the zygote will develop as female [62]. The absence of the CSD mechanism in *C*. *flavipes* implies that the parasitoid can be created by several inbred generations without showing the negative consequences of an increased proportion of males owing to the production of diploid males [33].

Although some significant differences between populations and over the generations have been observed in this study, these results were not sufficient to indicate deleterious effects of the inbreeding process. After forty years since the beginning of the biological control program with *C*. *flavipes* in Brazil, there is still little literature on the biology and role of this parasitoid in the field. Our results demonstrate the performance of this insect in the laboratory, but their activity and behavior were poorly documented both during its introduction in the country and over the years of its liberation. Thus, it is not possible to infer that their performance has been affected dramatically as a result of rearing methodology adopted by the mass production labs.

The inherent species characteristics such as type of sex determination, gregarious habit, and post-emergence behavior [63] lead us to believe that, indeed, this parasitoid is able to avoid the deleterious effects of inbreeding. However, the studies that support our results were performed with different strains from those introduced in Brazil and may differ in important biological characteristics of the insect.

This work highlights the need to conduct further studies seeking to ascertain through molecular analysis whether there has indeed been a loss in genetic variability of the introduced strain. Studies with larger numbers of strictly inbred generations must also be done in order to confirm the absence of deleterious effects. Field studies should be considered to evaluate the real effectiveness of this parasitoid or probable "domestication." Studies of Volpe et al. [64] demonstrate 26.5% loss in the ability to disperse in *C*. *flavipes* compared to those individuals initially introduced [65]. Comparison between the biology of parasitoids collected from different regions of Brazil would also help to determine if there are better adaptations of this strain in certain regions of the country.

Finally, based on the results, it can be suggested that quality control is standardized and performed periodically in laboratory mass-rearing facilities for *C*. *flavipes*, in order to maintain a record that enables the monitoring and development of colonies in the country. Consequent degenerative effects of inbreeding are not expressed in the biology and survival after 10 consecutive generations of strict inbreeding of *C*. *flavipes*.

Future studies should be conducted to compare the Brazilian population of *C*. *flavipes* with a population from their place of origin in the Indo-Australian region [66]. Thus, we would know whether the Brazilian population lost quality after it was reared in the laboratory for over 40 years. However, there are limitations to this study, particularly with respect to the Brazilian laws, which are too bureaucratic. The introduction of a species of parasitoid in Brazil may take up to a year, which makes it difficult to conduct this research. However, for the continued success of the biological control program for *C*. *flavipes* in Brazil, this question needs to be answered. Therefore, even with the difficulties, this study should be conducted in the near future.

## Supporting Information

S1 Appendix Flowchart of generations of *Cotesia flavipes*. See file S1_Appendix.pdf
(PDF)

## References

[1] Conab—Companhia Nacional de Abastecimento. Acompanhamento de safra brasileira: cana-de-açúcar. Safra 2014/2015. Primeiro levantamento, abril/2014—Companhia Nacional de Abastecimento.–Brasília: Conab 2014. Available: http://www.conab.gov.br/OlalaCMS/uploads/arquivos/14_04_15_15_44_37_boletim_cana_portugues_-_1o_lev_-_14.pdf. Accessed 20 May 2014.

[2] Unica—União da Agroindústria Canavieira de São Paulo. Coletiva de encerramento de safra 2014/2014 e projeções da safra 2014/2015. Available: http://www.unica.com.br/documentos/apresentacoes/. Accessed 07 May 2014.

[3] Garcia JF, Botelho PSM, Macedo LPM. Criação do Parasitoide Cotesia flavipes em laboratório. In: Bueno VHP, editor. Controle Biológico de pragas: produção massal e controle de qualidade. Lavras: UFLA; 2009. pp. 199–220.

[4] Pinto AS, Garcia JF, Botelho BSM. Controle biológico na cana-de-açúcar. In: Pinto AS, Nava DE, Rossi MM, Malerbo-Souza DT, editors. Controle Biológico de Pragas na Prática. Piracicaba: CP 2; 2006. pp. 65–74.

[5] Dinardo-Miranda LL. Pragas In: Dinardo-Miranda LL, Vasconcelos ACM, Landell MGA, editors. Cana-de-açúcar. Campinas: Instituto Agronômico; 2008 pp. 349–404.

[6] Marconato JR. Aspectos biológicos de Diatraea saccharalis (Fabr., 1794) (Lep., Pyralidae) em meio artificial contendo diferentes genótipos de sorgo e milho na forma de colmos secos e triturados. M.Sc. Dissertation, Faculdade de Ciencias Agrarias e Veterinarias, Universidade Estadual Paulista “Julio de Mesquita Filho”. 1988.

[7] Botelho PSM. Quinze anos de controle biológico de *Diatraea saccharalis* utilizando parasitoides. Pesqui Agropecu Bras. 1992;27: 255–262.

[8] Botelho PSM, Macedo N. Cotesia flavipes para o controle de Diatraea saccharalis. In: Parra JRP, Botelho PSM, Corrêa-Ferreira BS, Bento JMS, editors. Controle biológico no Brasil: parasitoides e predadores. São Paulo: Manole; 2002. pp. 409–425.

[9] Polanczyk RA, Almeida LC, Padulla L, Alves SB. Pragas de cana-de-açúcar x métodos alternativos de controle. Biotecnologia Cienc Desenvolv. 2004;33: 14–17.

[10] Hivizi CL, Bueno VHP, Silva AC, Carvalho LM. Controle de qualidade do parasitoide *Cotesia flavipes* In: Bueno VHP, editor. Controle Biológico de pragas: produção massal e controle de qualidade. Lavras: UFLA; 2009 pp. 371–380.

[11] Vacari AM, De Bortoli SA, Borba DF, Martins MIEG. Quality of *Cotesia flavipes* (Hymenoptera: Braconidae) reared at different host densities and the estimated cost of its commercial production. Biol Control. 2012;63: 102–106.

[12] Prezotti L, Parra JRP. Controle de qualidade em criações massais de parasitoides e predadores. In: Parra JRP, Botelho PSM, Corrêa-Ferreira BS, Bento JMS, editors. Controle biológico no Brasil—parasitoides e predadores. São Paulo: Manole; 2002. pp. 295–308.

[13] Riscado GM. Eficiência comparada de Apanteles flavipes (Cameron, 1891) no controle de Diatraea spp. no Rio de Janeiro. M.Sc. Dissertation, São Paulo University ESALQ/USP. 1982.

[14] Mackauer M. Genetic aspects of insect production. Entomophaga 1972;17: 27–48.

[15] Bartlett AC. Genetic changes during insect domestication In: King EG, Leppla NC, editors. Advances and challenges in insect rearing. Washington: USDA; 1984 pp. 2–8.

[16] Ralls K, Frankham R, Ballou JD. Inbreeding and outbreeding In: Levin SA, editor. Encyclopedia of Biodiversity. Oxford: Elsevier; 2007 pp 1–9.

[17] Charlesworth D, Charlesworth B. Inbreeding depression and its evolutionary consequences. Ann Rev Ecolog Syst. 1987;18: 237–268.

[18] Cassel A, Windig J, Nylin S, Wiklund C. Effects of population size and food stress on fitness–related characters in the Scare Heath, a rare butterfly in Western Europe. Conserv Biol. 2001;159: 1667–1673.

[19] Van Lenteren JC. Testes para o controle de qualidade de agentes de controle biológico comercializados In: Bueno VHP, editor. Controle Biológico de pragas: produção massal e controle de qualidade. Lavras: UFLA; 2009 pp. 339–370.

[20] Griffiths AJF, Wessler SR, Lewontin RC, Carroll SB. Introdução à genética. Rio de Janeiro: Guanabara Koogan; 2008.

[21] Littell RC, Milliken GA, Stroup WW, Wolfinger RD, Schabenberger O. SAS for mixed models 2nd ed. Cary: SAS Institute Inc; 2006.

[22] Kaplan EL, Meyer P. Nonparametric estimation from imcomplete observations. J Am Stat Assoc. 1958;53: 457–481.

[23] Henter HJ. Inbreeding depression and hapodiploidy: experimental measures in a parasitoid and comparisons across diploid and hapodiploid insect taxa. Evolution 2003;57: 1793–1803. 14503621 10.1111/j.0014-3820.2003.tb00587.x

[24] Crozier RH. Adaptive consequences of male haploidy In: Helle W, Sabelis MW, editors. Spider Mites: Their Biology, Natural Enemies and Control. Amsterdam: Elsevier Science Publishers; 1985 pp. 201–222.

[25] Werren JH. The evolution of inbreeding in haplodiploid organisms In: Thornhill NW, editor. The Natural History of Inbreeding and Outbreeding. Chicago: University of Chicago Press; 1993 pp. 42–59.

[26] Antolin MF. A genetic perspective on mating systems and sex ratios of parasitoid wasps. Res Popul Ecol. 1999;41: 29–37.

[27] Godfray HCJ, Cook JM. Mating systems of parasitoid wasps In: Choe JC, Crespi B, editors. The evolution of mating systems in insects and arachnids. Cambridge: Cambridge University Press; 1997 pp. 211–225.

[28] Godfray HCJ. Parasitoids: behavioral and evolutionary ecology Princeton: Princeton University Press; 1994.

[29] Tagawa J, Kitano H. Mating behaviour of the braconid wasp, *Apanteles glomeratus* L. (Hymenoptera: Braconidae) in the field. Appl Entomol Zool. 1981;16: 345–350.

[30] Gu HN, Dorn S. Mating system and sex allocation in the gregarious parasitoid *Cotesia glomerata*. Anim Behav. 2003;66: 259–264.

[31] Elias J, Dorn S, Mazzi D. Inbreeding in a natural population of the gragarious parasitoid wasp *Cotesia glomerata*. Mol Ecol. 2010;19: 2336–2345. 10.1111/j.1365-294X.2010.04645.x 20465585

[32] Arakaki N, Ganaha Y. Emergence pattern and mating behavior of *Apanteles flavipes* (Cameron) (Hymenoptera:Braconidae). Appl Entomol Zool. 1986;21: 382–388.

[33] Niyibigira EI, Overholt WA, Stouthamer R. *Cotesia flavipes* Cameron (Hymenoptera: Braconidae) does not exhibit complementary sex determination (ii) Evidence from laboratory experiments. Appl Entomol Zool. 2004;39: 717–725.

[34] Ridley M. Clutch size and mating frequency in parasitic Hymenoptera. Am Nat. 1993;142: 893–910.

[35] Cano MAV, Santos EM, Pinto AS. Produção de Cotesia flavipes para o controle da broca-da-cana. In: Pinto AS, editor. Controle de pragas da cana-de-açúcar. Sertãozinho: Biocontrol; 2006. pp. 1–64.

[36] Kazmer DJ, Luck RF. Field tests of the size-fitness hypothesis in the egg parasitoid *Trichogramma pretiosum*. Ecology 1995;76: 412–425.

[37] Charlesworth B, Morgan MT, Charlesworth D. Multilocus models of inbreeding depression with synergistic selection and partial self-fertilization. Genet Res. 1991;57: 177–194.

[38] Hernández D. Estudio de algunos aspectos biológicos de *Cotesia flavipes* (Cameron) (Hymenoptera: Braconidae) parasitoide de *Diatraea saccharalis* (Fabricius) (Lepidoptera: Crambidae). Entomotropica 2010;25: 69–81.

[39] Zhou Y, Gu H, Dorn S. Effects of inbreeding on fitness components of *Cotesia glomerata*, a parasitoid wasp with single-locus complementary sex determination (sl-CSD). Biol Control 2007;40: 273–279.

[40] Wiedenmann RN, Smith JW. Parasitization of *Diatraea saccharalis* (Lepidoptera: Pyralidae) by *Cotesia chilonis* and *C*. *flavipes* (Hymenoptera: Braconidae). Environ Entomol. 1995;24: 950–961.

[41] Ma W, Kuijper B, De Boer JG, Van De Zande L, Beukeboom LW, Wertheim B, et al Absence of complementary sex determination in the parasitoid wasp genus *Asobara* (Hymenoptera: Braconidae). PloS ONE 2013;8: e60459 10.1371/journal.pone.0060459 23637750 PMC3614920

[42] Stouthamer R, Luck RF, Werren JH. Genetics of sex determination and the improvement of biological control using parasitoids. Environ Entomol. 1992;21: 427–435.

[43] Zhou Y, Gu HN, Dorn S. Single-locus sex determination in the parasitoid wasp *Cotesia glomerata* (Hymenoptera: Braconidae). Heredity 2006;96: 487–492. 16622470 10.1038/sj.hdy.6800829

[44] Stouthamer R, Luck RF, Werren JH. Genetics of sex determination and the improvement of biological control using parasitoids. Environ Entomol. 1992;21: 427–435.

[45] Whiting PW. Multiple alleles in complementary sex determination of *Habrobracon*. Genetics 1943;28: 365–382. 17247094 10.1093/genetics/28.5.365 PMC1209216

[46] Snell GD. The determination of sex in *Habrobracon*. Proc Natl Acad Sci USA 1935;219: 446–453. 10.1073/pnas.21.7.446 PMC1076623 16587997

[47] Crozier RH. Heterozygosity and sex determination in haplo-diploidy. Am Nat 1971;105: 399–412.

[48] Cook JM, Crozier RH. Sex determination and population biology in the Hymenoptera. Trends Ecol Evol 1995;10: 281–286. 21237037 10.1016/0169-5347(95)90011-x

[49] Bull JJ. Coevolution of haplo-diploidy and sex determination in the Hymenoptera. Evolution 1981;35: 568–580. 28563595 10.1111/j.1558-5646.1981.tb04918.x

[50] Petters RM, Mettus RV. Decreased diploid male viability in the parasitic wasp *Bracon hebetor*. J Heredity 1980;71: 353–356.

[51] Smith SG, Wallace DR. Allelic sex determination in a lower hymenopteran, *Neodiprion nigroscutum* Midd. Can J Genet Cytol 1971;13: 617–621.

[52] El Agoze M, Drezen JM, Renault S, Periquet G. Analysis of the reproductive potential of diploid males in the wasp *Diadromus pulchellus* (Hymenoptera: Ichneumonidae). Bull Entomol Res. 1994;84: 213–218.

[53] MacBride DH. Failure of sperm of *Habrobracon* diploid males to penetrate the eggs. Genetics 1946;31: 224. 21021055

[54] Torvik MM. Genetic evidence for diploidisin of biparental males in *Habrobracon*. Biol Bull. 1931;61: 139–156.

[55] Niyibigira EI, Overholt WA, Stouthamer R. *Cotesia flavipes* Cameron and *Cotesia sesamiae* (Cameron) (Hymenoptera: Braconidae) do not exhibit complementary sex determination: evidence from field population. Appl Entomol Zool. 2004;39: 705–715.

[56] Michel-Salzat A, Whitfield JB. Preliminary evolutionary relationships within the parasitoid wasp genus *Cotesia* (Hymenoptera: Braconidae: Microgastrinae): combined analyses of four genes. Syst Entomol. 2004;29: 371–382.

[57] Antolin MF, Strand MR. Mating system of *Bracon hebetor* (Hymenoptera: Braconidae). Ecol Entomol. 1992;17: 1–7.

[58] Crozier RH. Evolutionary genetics of the Hymenoptera. Ann Rev Entomol. 1977;22: 263–288.

[59] Elias J, Mazzi D, Dorn S. No need to discriminate? Reproductive diploid males in a parasitoid with complementary sex determination. PLoS ONE 2009;4: e6024 10.1371/journal.pone.0006024 19551142 PMC2696080

[60] De Boer JG, Ode PJ, Rendahl AK, Vet LEM, Whitfield JB. Experimental support for multiple-locus complementary sex determination in the parasitoid *Cotesia vestalis*. Genetics 2008;180: 1525–1535. 10.1534/genetics.107.083907 18791258 PMC2581954

[61] De Boer JG, Kuijper B, Heimpel GE, Beukeboom LW. Sex determination meltdown upon biological control introduction of the parasitoid *Cotesia rubecula*. Evol Appl. 2012;5: 444–454. 10.1111/j.1752-4571.2012.00270.x 22949920 PMC3407863

[62] Poirié M, Periquet G, Beukeboom LW. Arrhenotoky, the hymenopteran way of determining sex. Sem Cell Dev Biol. 1992;3: 357–361.

[63] Joyce AL, Bernal JS, Vinson SB, Hunt RE, Schulthess F, Medina RF. Geographic variation in male courtship acoustic and genetic divergence of populations of the *Cotesia flavipes* species complex. Entomol Exp Appl. 2010;137: 153–164.

[64] Volpe HXL, Barbosa JC, Viel SR, Goulart RM, Vacari AM, Salas C, et al Determination of method to evaluate parasitism and cover area for studies on *Cotesia flavipes* in sugarcane. Afr J Agric Res. 2014;9: 436–447.

[65] Botelho PSM, Macedo N, Mendes AC. Aspects of the population dynamics of Apanteles flavipes (Cameron) and support capacity of its host Diatraea saccharalis (Fabr.). In: Congress of the International Society of Sugar Cane Technologists, 17, Proceedings\ ISSCT. Manila, Philippines; 1980.

[66] Polaszek A, Walker AK. The *Cotesia flavipes* species-complex: parasitoids of cereal stem borers in the tropics. Redia 1991;74: 335–341.

