# Supplementary figures and images for: Quality of the Exotic Parasitoid Cotesia flavipes (Hymenoptera: Braconidae) Does Not Show Deleterious Effects after Inbreeding for 10 Generations
Source: PLoS One. 2016 Aug 10;11(8):e0160898. doi: 10.1371/journal.pone.0160898 (PMC4979891; doi:10.1371/journal.pone.0160898)

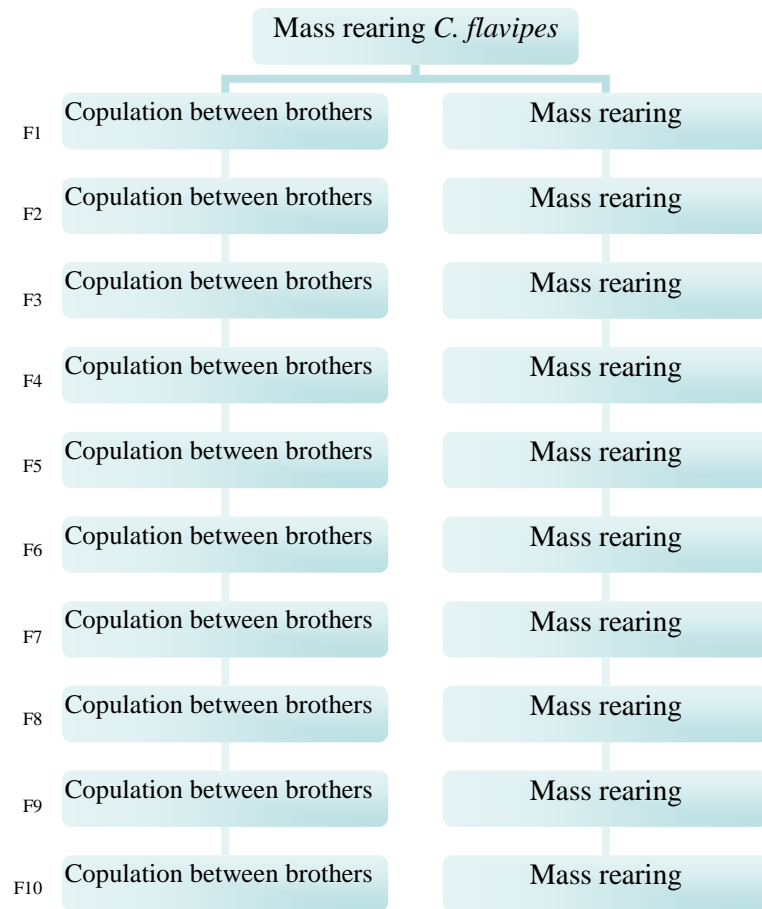

**Figure 1.** Flowchart of generations of *Cotesia flavipes*.

Supplement: S1 Appendix — See file S1_Appendix.pdf (PDF) [file pone.0160898.s001.pdf]
